# Adverse Effects of Smartphone Addiction among University Students in South Korea: A Systematic Review

**DOI:** 10.3390/healthcare11010014

**Published:** 2022-12-21

**Authors:** Chiara Achangwa, Hyun Sik Ryu, Jae Kwang Lee, Ju-Dong Jang

**Affiliations:** 1 Department of Public Health and Welfare, The Graduate School, Konyang University 709 Ho, Myeongkok Medical Building 158, Gwanjeodong-ro, Seo-gu, Daejeon 35365, Republic of Korea; 2 Department of Emergency Medicine, Konyang University Hospital, 685 Gasuwon-Dong Seo-gu, Daejeon 35365, Republic of Korea

**Keywords:** adverse effects, smartphone addiction, university students, systematic review, Korea

## Abstract

Background: Globally there has been an exponential increase in the penetration of smartphones among the youth population and smartphones have become indispensable in the daily lives of university students in South Korea. Several studies have associated the problematic use of smartphones or addiction with different adverse outcomes. The goal of this study was to collate empirical evidence and provides an overall synthesis of the literature about the adverse effects of smartphone addiction on university students in South Korea. Method: We carried out a systematic review of the published literature between August and October 2022 on the adverse effects of smartphone addiction on university students in South Korea, published between 2012 and 2022 in Pubmed/Medline, PsycInfo, Embase, Biomed-Central, Web of Science, Directory of Open Access Journals, Elsevier’s collection, Wiley Online Library, SpringerLink, Sage Journal’s collection and Cochrane Library. Results: Thirty-four articles published between 2012 and 2022 were included in the synthesis of this review. Eight studies explored the association between smartphone addiction and the psychological and mental health of university students in Korea. Smartphone addiction was associated with physical health leading to sleep disorders and musculoskeletal and neurological problems. Academic performance, procrastination, impulsivity, self-esteem, reduced social interaction, solitude, and suicide were also negatively associated with smartphone addiction. Conclusions: Our study adds to the literature regarding the adverse effects of smartphone addiction on university students in Korea and provides more information for addiction prevention and health promotion activities.

## 1. Background

Globally the use of smartphones has exponentially increased becoming an inherent part of many individuals’ daily lives and has also introduced various changes in our daily activities and habits [1]. This exponential increase is because, in addition to the phone and text services provided by conventional mobile phones, smartphones contain new technology that provides an interface to make real-time broadcasts, access to a wide range of contents, and send/receive emails [2]. 

As of 2019, the global smartphone penetration was reported to be approximately 41.5% of the global population [3]. In 2014, the number of smartphone users in South Korea was reported to be 39 million. However, currently, the number of smartphone users is estimated to be around 53.5 million, accounting for 97% of the Korean population. Among this population, it is reported that individuals between the ages of 20–30 years have a 100% penetration rate [4]. Due to this increase in the smartphone penetration rate in the population, research shows that excessive smartphone use is problematic [5,6,7,8,9,10]. In the literature, the terms “problematic mobile or smartphone use” [11], “Smartphone addiction” [12,13,14,15] “smartphone dependence” [16,17,18], and “smartphone overuse” [8,19], have all been used interchangeably to express more or less the same phenomenon, which is, an individual’s inability to regulate their use of the smartphone, resulting in negative consequences in daily life.

In addition, due to the multifaceted nature of smartphone addiction, its definition still remains controversial [20,21,22]. However, according to Lee et al., smartphone addiction is a disorder involving the compulsive pathological overuse of smartphone devices. Because smartphone devices are used to access and use the internet, the term internet addiction has also been used to express some form of smartphone addiction [21]. According to Ismail et al., smartphone addiction does not differ from internet addiction in that they share common pathological manifestations [23]. Nevertheless, the standard cut-off point for determining when smartphone use has become an addiction is yet to be determined. 

Smartphone addiction could be facilitated by easy access to the internet, and other popular social media apps such as Facebook, YouTube, Instagram, Twitter and other platforms that are not only designed in ways that increase the amount of time people spend on them but also monetize activities on the apps resulting in the continuous need to be online which has led to a new syndrome known as “Fear of Missing Out (FoMO)”. FoMO refers to a state in which individuals spend much of their time on social media with the fear of missing out on the latest information in social networks [24]. This desire to always be online and be up to date with information and communication has resulted in nomophobia which is defined as “the fear of lacking communication, not having contact with the mobile phone or an uncontrollable fear of leaving home without a phone [25]. This has been reported to have various health and cognitive implications [20]. Even though, some studies suggest a direct relationship between smartphone addiction and nomophobia, the relationship between them is not very clear [26]. Moreover, there are existing debates as to whether “smartphone addiction” ought to be determined based on quantity, patterns of use, or by the negative consequences of its use [27,28,29].

Attempts have been made to explain the several routes to smartphone addiction including the ability to regulate self-control and emotions, impulsivity, self-esteem, neuroticism, and distorted thinking [30,31,32]. However, based on different demographics (gender, age, socio-economic status) and personality (introvert/extrovert) characteristics individuals, have different triggers that make them use their smartphones in a problematic way [33,34].

University students are in the age group most interested in possessing smartphones for many different reasons [35]. They use smartphones for a variety of activities such as studying, entertaining, accessing the internet or social networks, and social communication [32]. Despite the many benefits of smartphone use, empirical research suggests that individuals are addicted to or overly dependent on smartphones, resulting in negative consequences affecting their health [20], safety [36,37,38], and daily lives [25]. The empirical literature suggests that there exist direct and indirect associations between smartphone addiction and health, and other related issues among university students in Korea [39]. 

After a thorough review of previous studies on smartphone addiction among university students in Korea, several issues were identified. First, there were varying reports on the negative outcomes of smartphone addiction among university students. Second, the use of different tools to evaluate smartphone addiction and its effects among university students. The literature also revealed that there is no consensus among these studies regarding the adverse effects of smartphone addiction on university students in South Korea. 

Therefore, this systematic review aimed to provide an overall synthesis of the literature regarding the adverse effects of smartphone addiction on university students in South Korea. 

## 2. Methods

**Figure 1:**
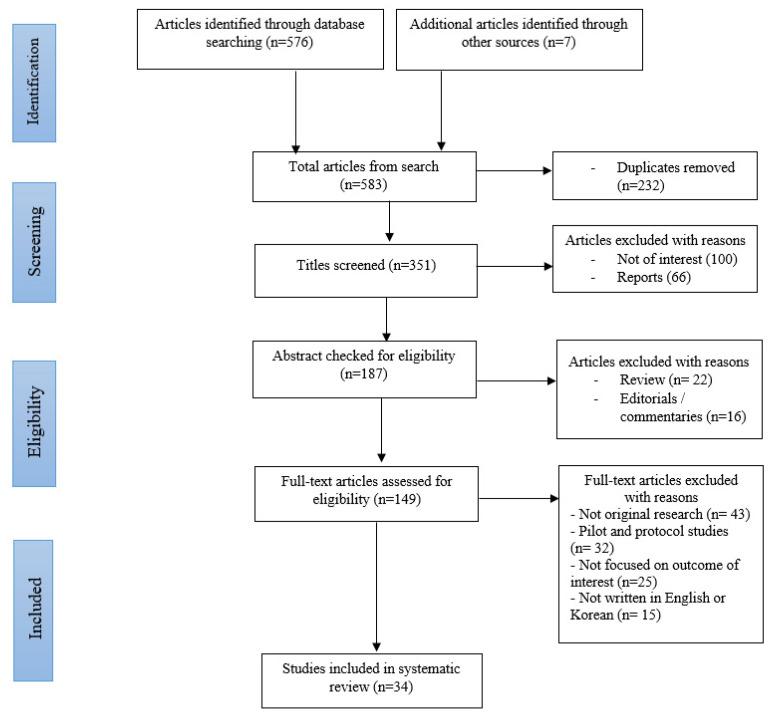
PRISMA Flow diagram showing the selection of review articles.

We conducted a systematic review of already published literature from 1 January 2012 to 1 October 2022. Article collection and synthesis were done following the Preferred Reporting Items for Systematic Reviews (PRISMA) guidelines (Figure 1) [40].

We searched articles from major databases including Pubmed/Medline, PsycInfo, Embase, Biomed-Central, Web of Science, Directory of Open Access Journals, Elsevier’s collection, Wiley Online Library, SpringerLink, Sage Journal’s collection, and Cochrane Library. In addition, we collected additional articles from the secondary references of included studies. The databases were searched between August and October 2022. Articles were included if they were original articles with full introductions, methods, results, and discussion sections, published in a peer-reviewed journal between 2012–2022, and focused on the effect of smartphone overuse or addiction on university students in Korea. Published articles in two different languages were used: English and Korean languages (languages in which the authors are proficient). Studies were excluded if they only examined internet addiction but not in the context of smartphone use, articles focusing on the positive effects of smartphones, and if they were reviews, editorials, conference proceedings, dissertations, or commentaries without primary data or peer review. A set of search terms were created with truncations, Medical Subject Headings (MESH), and Boolean operators, as shown in Table 1. These terms were also used in Korean, to search for articles that were published in the Korean language. 

The titles and abstracts of all included articles were reviewed independently by two authors (A.C., J.K.L.) against the above inclusion and exclusion criteria. Inter-rater reliability was calculated using Cohen’s Kappa. The full papers of all studies included after abstract screening were retrieved and reviewed for a second time based on the inclusion and exclusion criteria by the same two authors (A.C., J.K.L.). Disputes at either stage were resolved through discussion, with 2 other authors (H.S.R., J-N.J.) where necessary, until consensus was reached. 

## 3. Results

### 3.1. Study Characteristics

At the end of the article search from the databases, a total of 583 potential studies were identified. The final sample consisted of 34 articles. Inter-rater reliability at the screening phase was κ = 0.91 (95% CI 0.88–0.94), representing a good alignment. All studies took place primarily in South Korea with the study participants recruited from a range of universities. Smartphone addiction was directly or indirectly negatively associated with different outcomes. A heterogenous use of different tools, methods, and smartphone addiction scales was noted across the included studies. The Smartphone Addiction Scale was used in four of the studies, and the Smartphone Addiction Proneness Scale (SAPS) in two of the included studies. The remaining 28 studies each used different tools and measures. 

### 3.2. Main Findings

#### 3.2.1. Psychological and Mental Health

Smartphone addiction was reported to impact the psychological and mental health of university students in 11 studies conducted in Korea. In a study by Kim et al., students who reported poor health were significantly associated with smartphone addiction (Odds ratio (OR) = 1.98; 95% CI = 1.22–3.21). In addition, the study showed that the students with stress-related symptoms were significantly associated with smartphone addiction, depicting an approximately twofold increased risk compared to those without these symptoms [8]. Another similar study revealed that university students with smartphone addiction had higher psychiatric symptom scores compared to their counterparts with no addiction [41]. Smartphone addiction was mostly associated with anxiety and depression which are components of mental health [8,12,13,41,42,43,44,45].

#### 3.2.2. Physical Health

##### Poor Sleep Quality

Ten studies reported that smartphone addiction was associated with poor sleep quality in university students in South Korea. Choi et al. noted that high mobile phone addiction was correlated with poor sleep quality [46]. Another study reported that higher levels of smartphone addiction and stress were associated with lower sleep quality [47]. In addition, Kwon et al., also reported that attention-deficit hyperactivity disorder (ADHD) symptoms were positively correlated with smartphone addiction (*p* < 0.01) and poor sleep quality (*p* < 0.01) [9]. The risk of sleep problems was shown to increase in the addiction proneness groups (OR = 1.99; 95% CI: 1.33–2.98) than in the normal-user groups [48]. Many other studies reported a correlation between smartphone addiction and the quality of sleep among university students in South Korea [49,50,51,52,53].

##### Musculoskeletal and Neurological Problems

The extensive use of smartphones is reported to be associated with physical health–related problems, including pain in the shoulders, back, neck, legs, and wrists. According to Kim et al., back pain was found to be positively correlated with the size of the smartphone’s liquid crystal display (LCD) screen, and pain in legs and feet was found to have a negative correlation with the length of time that the smartphone was used [54]. According to a study by Paek, smartphone overuse was positively associated with dry eye syndrome (*p* < 0.001), neck pain (*p* < 0.05), and hand pain (*p* < 0.05) [55]. In addition, uncontrollable use of smartphones for long periods exposes hands to intense stresses that may lead to pain and musculoskeletal disorders of the neck, hand, and thumb [56]. Because of the nature of smartphones, individuals usually hold the device with a single hand, which forces only the thumb to use the keys resulting in pain [57]. 

##### Accidents

Two studies reported on the association between smartphone addiction and accidents. According to a study by Kwon et al., out of the 441 students who participated in the study, 57.9% experienced accidents or near misses when using smartphones while walking [58]. Another study by Kim et al., reported that compared with normal users, participants who were addicted to smartphones were more likely to experience accidents (OR = 1.90, 95% CI: 1.26–2.86), falling from height/slipping (OR = 2.08, 95% CI: 1.10–3.91), and bumps/collisions (OR = 1.83, 95% CI: 1.16–2.87) [15]. 

#### 3.2.3. Effect on Academic Performance

While some studies in the literature reported that smartphone addiction had a negative effect on the academic performance of university students, others reported no effect. Winskel et al., reported that there were no significant correlations found between problematic smartphone addiction and grade point average (GPA) scores [59]. Han et al. used different models to show how smartphone addiction and college students’ behavioral intention influences academic performances by study subjects [60]. Lee and Lee found that an increase in the daily total smart device use time negatively affected students’ GPA [61]. It was also found that students with smartphone addiction were constantly interrupted by applications on their phones when they are studying and do not have enough control over their smartphone learning plan and its process [2]. 

#### 3.2.4. Procrastination and Impulsivity

There was also a direct relationship between impulsivity and smartphone addiction [62]. In addition, university students with ADHD symptoms had difficulties coping with repeated cycles of negative thoughts and worries, irregular lifestyles due to poor time management, dissatisfaction with academic performance and interpersonal relationships, self-dissatisfaction, and decreased self-esteem [63].

#### 3.2.5. Self-Esteem, Reduced Social Interaction, Solitude, and Suicide

Several studies have outlined the impact of smartphone addiction on various levels of human interactions and relationships [64,65,66,67]. The smartphone addiction and human relationship health results were negatively correlated (*p* = 0.011). However, communication ability and human relationship health results were positively correlated (*p* < 0.001) [68]. Smartphone addiction was negatively associated with interpersonal relationships [69,70]. Jeong et al. reported an association between internet, gaming, or smartphone addiction and suicidal ideation [71]. A summary of all the included studies is found in Table 2.

## 4. Discussion

The main aim of this systematic review was to identify and provide an overall synthesis of literature regarding the adverse effects of smartphone addiction on university students in South Korea. To the best of our knowledge, this present review is the first review that collates all the adverse outcomes of smartphone addiction among university students in Korea. In the past decade, smartphone penetration has greatly increased among youth groups, especially among university students, and the problem of addiction is a serious rising public health problem. Therefore, our review provides a timely and comprehensive evidence base for designing corresponding intervention measures to inform and address the adverse outcomes of smartphone addiction among university students in South Korea. 

Previous literature highlighted existing discrepancies in smartphone addictive use by gender [46], the field of study [13], and level of education [8] among university students in South Korea. The main differences in these groups are the patterns of use, motivations, or purposes. However, a mixed-approach investigation including both quantitative and qualitative methods is recommended to provide a comprehensive understanding of all the facets of smartphone addiction and its impact on university students in Korea.

Throughout the reviewed literature, smartphone addiction was consistently associated with decreased psychological and mental health including stress, anxiety, and depression in university students in Korea. Due to the exponential increase in smartphone penetration rate among university students, the incidence of mental health conditions has also increased representing a significant burden on the healthcare system and a growing public health problem that should be of great concern not only to policymakers in Korea but the parents as well.

This was similar to studies in different countries, which have stated an increase in the incidence of smartphone addiction associated with mental health issues [69,70,71]. Studies have also shown that compared to the older population, younger populations are more vulnerable to developing mental conditions and harmful long-lasting behaviors and addictions which can shape their subsequent life course [72]. Therefore, policies should be developed to address and prevent the possible long-term harmful impact of smartphone addiction on future generations’ mental health and well-being.

Another aspect of concern was the impact of smartphone addiction on physical health including musculoskeletal and neurological problems, accidents, and sleep quality among university students in Korea. The poor sleep quality, reduced sleep duration, and more frequent disturbances reported could be a result of the use of different interactive apps including various social media, and gaming apps [73]. In line with these findings, smartphone addiction has also been reported to be associated with physical health in university students in different countries [74,75].

While empirical literature reports some positive impacts of smartphone use among students, its overuse has been reported to have a direct negative effect on academic performance. In this review, smartphone addiction was negatively associated with educational outcomes among university students implying that the greater the use of smartphones while studying, the greater the negative impact on learning. Kim et al., [15] reported that students had difficulty controlling how and when to use their smartphones, impacting their study times. To address this rising phenomenon, policymakers need to implement various cognitive-behavioral therapies, and interventions, among university students. In addition, due to the high penetration rate of smartphones among university students, educators should implement guidelines to identify the times when smartphones can or cannot be used by students in higher institutions and could also implement their use to support the curriculum [76].

Consistent with other studies [77,78], we found that smartphone addiction had an impact on the level of social interaction of university students. These students even in the physical presence of their friends and/or family have a strong desire to use their smartphones, and most often results in a lack of focus, engagement, and real-time involvement with their counterpart which consequently plays a negative role in interpersonal communications and connections, thereby causing considerable negative effects on relationships and social interactions [79,80]. In addition, during the COVID-19 period and due to the prolonged lockdown period, most lectures were switched from face-to-face to online lectures in which some students use their smartphones as well as other smart devices. This resulted in university students spending time away from their peers and educators, leading to solitude and reduced social interaction.

It is worth noting that, in this review, the reported adverse effects (depression, procrastination, health problems, self-esteem problems) found in the current study could be symptoms or antecedents of smartphone addiction rather than the consequences. For example, smartphones can act as a refuge for individuals already presenting symptoms of the above adverse effects. As such, they may be a medium that amplifies these symptoms rather than causes them. Similar outcomes have been observed in students’ social media usage where social media was not the cause of depression but rather depression was a contributing factor to social media usage [81].

Another significant direction that could be worthy of note is the impact of parenting on how students use their smartphones. Previous research in Korea has shown that, some parents to relieve themselves of parental emotional and physical exhaustion, expose their children very young to the use of smartphones [82]. Additionally, due to physical and mental immaturity, these children cannot regulate their use times and ways of use growing to be dependent on their smartphones, taking this same problematic behavior to the university [83]. However, further detailed research using a causal study design is required to properly investigate the effect of parenting on the development of smartphone addiction among university students in South Korea.

The major strength of this review is that it provides a comprehensive synthesis of the adverse effects of smartphone phone addiction among university students in South Korea. However, the authors of this study observed some limitations in the studies included in this review. Firstly, the studies used in this review all used cross-sectional data and conducted either a correlational synthesis or linear or logistic regression analyses, hence the results cannot be given a causal interpretation. To advance empirical knowledge in the literature, well-designed longitudinal and interventional research is essential for addressing the adverse effects of smartphone addiction among university students. Secondly, for all the adverse effects recorded in this study, there was a lack of research investigating theoretical mechanisms of how smartphone addiction causes these effects. Providing more detailed information on the mechanisms at work could be important in the design of policy. To implement effective and reliable policy measures concerning smartphone addiction among university students, it will be helpful to know what precisely causes the relationship between smartphone addiction and its adverse effects. Thirdly, our focus was mainly on university students limiting the application of these findings to other populations. We recommend that future research should carry out longitudinal studies to investigate the detailed relationship between smartphone addiction and different adverse outcomes. 

## 5. Conclusions

This review shows that university students in South Korea are at risk of addiction to smartphone use. Despite heterogeneity in the reviewed studies, the studies suggest poor physical health, psychological and mental health, poor academic performance, procrastination and impulsivity, reduced social interaction, solitude, and suicide are the most observed adverse effects of smartphone addiction among university students in South Korea. These findings from this review suggest the need for larger studies that will explore different dimensions (parental, personal, institutional, and social) of smartphone addiction among university students in South Korea. This review provides significant information to parents, educators, and policymakers. 

## Figures and Tables

**Table 1:** Search terms and linkage for the study.

Term Number	Term and Boolean Operator
Term 1 (Participants)	‘University students’ OR ‘College students’ OR ‘Students’ OR ‘Medical college students’ OR ‘Medical students’ OR ‘Nursing students’ OR ‘International students’ OR ‘Young Adults’ OR ‘Young students’ OR ‘Adult’ OR ‘Korea’ OR ‘South Korea’
	AND
Term 2 (Exposure)	‘Smartphone overuse’ OR ‘Smartphone use’ OR ‘Addiction’ OR ‘Smartphone addiction’ OR ‘Smartphone attachment’ OR ‘Problematic smartphone use’ OR ‘Excessive phone use’ OR ‘Mobile phone addiction’ OR ‘Cellular phone use’ OR ‘Mobile phone use’ OR ‘Cell phone use’
	AND
Term 3 (Outcomes)	‘Nomophobia’ OR ‘Effects’ OR ‘Adverse effects’ OR ‘Negative effects OR ‘Impact’ OR ‘Mental health’ OR ‘Mental disorder’ OR ‘Depression’ OR ‘Anxiety’ OR ‘Sleep’ OR ‘Psychological’ OR ‘Problems’ OR ‘Dependence’ OR ‘Pathology’ OR ‘Compulsive disorder’

**Table 2:** Summary table of the studies included in the systematic review.

Main Findings	Author, Year, and Location of Study	Study Details	Main Findings
Psychological and mental health	Kim et al., 2016; Korea [8].	Using a total of 608 college students, authors through a questionnaire-based online survey, investigated whether perceived psychological and subjective health status were related to smartphone overuse among Korean college students from August to September of 2016.	Students with smartphone overuse were significantly associated with an increased risk of psychological symptoms (*p* < 0.05). The stressed participants were 2.2 times more likely to overuse smartphones than those who reported little stress (OR ¼ 2.19; 95% CI ¼ 1.55–3.10). Similarly, the ORs (95% CIs) of smartphone overuse in depression symptoms and suicidal ideation were estimated as 1.91 (1.27–2.86) and 2.2 (1.52–3.31), respectively.
	Im et al., 2013; Korea [41].	A survey was carried out using two hundred and thirteen university students. Data was collected from 5–9 December 2011 in South Korea using the smartphone Addiction Scale.	Addicted scores were positively correlated with psychiatric symptom scores. Obsessive-compulsive score was the most highly correlated with addiction scores. There were significant differences in psychiatric symptom scores by group.
	Yoo et al., 2015; Korea [13]	A cross-sectional, descriptive correlational design was carried out in a sample of 378 undergraduate students to examine the differences in depression and smartphone addiction among four styles of perceived parenting in Korea.	There was a significant association between Levels of depression and smartphone addiction in all parenting groups. Students who perceived that both fathers and mothers were low in care, warmth, and support; but high in overprotection, control, and intrusiveness were vulnerable to depression and smartphone addiction.
	Kim et al., 2020; Korea [42]	Through a questionnaire-based study, the authors aimed at identifying the relationship between perceived smartphone addiction and physical and mental health among 314 female college students from 9 April to 31 May 2019.	The perceived smartphone addiction group had significantly more mental health problems (*p* < 0.001) and physical health problems (*p* = 0.001) compared to those without smartphone addiction.
	Yun et al., 2018; Korea [43]	The authors sought to understand the mental health and physical health problems of 261 college students caused by smartphone addiction.	Results showed that the smartphone addiction group had significantly higher scores in life stress (*p* < 0.001), depression (*p* < 0.001), social avoidance, and distress (*p* = 0.045) than the general user group.
	Dan et al., 2015; Korea [44]	Between 20 May 20 and 23 June 2014, authors investigated the relationship between smartphone addiction, physical symptoms, and psychological well-being in 214 nursing students.	Smartphone addiction was significantly associated with a higher frequency of psychological (*p* = 0.018) and physical symptoms (*p* < 0.001).
	Jeong et al., 2016; Korea [12]	The authors conducted a study to verify the relationships between mental health and Smartphone Addiction in 190 college students at a university in Seoul.	Results showed that smartphone addiction was significantly associated with mental health-related symptoms (*p* < 0.001).
	Choi et al., 2012; Korea [45]	The authors surveyed the status of smartphone usage and identified the influence of smartphone addiction on mental health, campus life, and personal relations of university students in Korea.	Authors found that smartphone addiction syndrome caused mental health, campus life, and personal relations problems in many ways (*p* < 0.05). In addition to significant direct effects, smartphone addiction has significant indirect effects on personal relations via mental health and campus life as a medium.
Physical health			
Poor sleep quality	Choi et al., 2015; Korea [46]	A cross-sectional study was carried out on 269 college students using a structured questionnaire to investigate physical activity level, sleep quality, attention control, and self-regulated learning along with smartphone addiction level among college students.	The results showed significant differences in smartphone addiction level and, gender, grade level, daily use time, physical activity level, sleep quality, and attention control. Smartphone addiction level had correlations with physical activity level, sleep quality, attention control, and self-regulated learning.
	Kim et al., 2019; Korea [47]	From 1 to 14 August 2018 using an online self-report survey, authors investigated the level of smartphone addiction and stress among university students and estimated the effects of those variables on sleep quality	Higher levels of smartphone addiction and stress were associated with lower sleep quality (*p* = 0.001).
	Kwon et al., 2022; Korea [9]	Through a cross-sectional study design, the authors examined the relationship between attention-deficit hyperactivity disorder ((ADHD)) symptoms, smartphone addiction, and poor sleep quality in university students between March and June 2017.	ADHD symptoms were positively correlated with smartphone addiction (*p* < 0.01) and poor sleep quality (*p* < 0.01).
	Min et al., 2017; Korea [48]	Through an online survey, with responses from 608 university students’ authors investigated the association between smartphone addiction proneness and sleep problems in Korean university students.	The addiction proneness groups had a higher Pittsburgh Sleep Quality Index (PSQI) score than the normal-user group (7.5 vs. 6.7, *p*-value < 0.0001). After adjustment for potential covariates (i.e., age, income, and smoking), PSQI scores were significantly increased in the addiction proneness groups (Beta coefficient = 0.69; 95% CI: 0.29~1.09). The risk of sleep problems was increased in the addiction proneness groups (odds ratio = 1.99; 95% CI: 1.33~2.98) than in the normal-user groups.
	Choi S, 2019; Korea [49]	A descriptive study involving 250 nursing students was carried out to investigate the relationship between Smartphone usage, sleep patterns, and nursing students’ learning engagement at a university in Chungbuk.	Quality of sleep and smartphone addiction were negatively correlated (*p* = 0.013).
	Ahn, S.Y. and Kim, Y.J. 2015; Korea [50]	Authors carried out a correlational descriptive study aiming to understand the relationship between smartphone use, stress, and quality of sleep, and the influence of smartphone use and stress on the quality of sleep in 738 nursing students	Smartphone use and quality of sleep were significantly correlated, where the higher the smartphone use, the lower the quality of sleep (*p* = 0.000).
	Heo et al., 2015; Korea [51]	The authors carried out a cross-sectional study to investigate any correlation between smartphone addiction and quality of sleep among graduate school students in Korea.	The result of this study revealed that the deeper the addiction to smartphones the lower the quality of sleep, especially in trouble staying awake.
	Jo et al., 2019; Korea [52]	This study was conducted between 22–27 February 2014 to identify the correlation between smartphone addiction, sleep quality, and depression in 304 college students.	The results showed that there was a significant negative correlation between smartphone addiction and sleep quality, a significant positive correlation between smartphone addiction and depression, and a significant negative correlation between sleep quality and depression (*p* < 0.05).
	Park JH 2019; Korea [53]	A descriptive research study was used to determine the effects of smartphone addiction on sleeping time and sleep deprivation among 280 health science college students.	There were correlations between smartphone addiction and students’ sleep deprivation (*p* = 0.005) but no correlations between their smartphone addiction and sleeping time. Furthermore, college students’ sleep deprivation was influenced by their smartphone addiction (*p* = 0.022).
	Kim et al., 2020; Korea [54]	From February to November 2014, through a cross-sectional study, a questionnaire was administered to 1060 Korean college student smartphone users. The authors investigated the relationships between sleep-related habits, inattention, and smartphone addiction tendency (SAT), and determined how chronotype mediates these factors in Korean college students.	More social jetlag and smartphone use before bedtime had both direct and indirect relationships with SAT. Participants with shorter exposure to sunlight and more anxiety symptoms had a direct relationship with (SAT *p* < 0.001).
Musculoskeletal and Neurological Problems	Kim et al., 2015; Korea [55]	The authors investigated the use of smartphones by 292 university students in selected areas, their musculoskeletal symptoms, and the associated hazard ratio. The study involved the completion of a self-administered questionnaire by dental hygiene students in Seoul, Korea	Results demonstrated that the most painful body regions after the use of smartphones were found to be the shoulders and neck. In the musculoskeletal system, back pain was found to have a positive correlation with the size of the smartphone’s liquid crystal display (LCD) screen (*p* < 0.05), and pain in legs and feet was found to have a negative correlation with the length of time that the smartphone was used (*p* < 0.05).
	Paek K. S.; 2017 Korea [56]	The authors collected data from 286 college students to identify the correlation between dry eye syndrome, upper extremity pain, depression, and addictive smartphone use among college students.	Results showed that dry eye syndrome (*p* < 0.001), neck pain (*p* < 0.05), hand pain (*p* < 0.05), and depression (*p* < 0.001) were positively related to addictive smartphone use.
	Lee, Hae-jung, 2016; Korea [57].	Seventy-eight university student volunteers, aged between 18 and 30 years (mean age 23.2), were assessed for: a head-neck posture by measuring cranial vertical angle, neck range of motions using a cervical range of motion device, and a deep neck flexor endurance using a stabilizer. Finally, subjects were asked about their neck pain and completed disability questionnaires.	The relationship between the smartphone usage time and usual pain intensity, neck-specific disability score, and affected days was positive, indicating that if subjects had longer usage time with their smartphone, they tended to have worse pain than usual (*p* = 0.04), more difficult in neck-specific activities (*p* = 0.04), and perceive more days disturbed in their daily activities (*p* < 0.01). Whereas the smartphone usage time was negatively related to left and right-side flexion ranges and neck location in a neutral sitting posture, which means if subjects spent longer time on a smartphone, they had limited right and left flexion ranges (*p* = 0.03, and *p* = 0.01, respectively) and tended to sit upright (*p* < 0.01).
	Kim et al., 2015; Korea [58]	From 21 April 2014 to 27 April 2014, authors carried out a study to investigate musculoskeletal pain regions and how to prevent pain and malfunction of the hand by correcting the angle of holding a smartphone.	Results showed that grip strength and pain score were significantly higher in the right arm compared to the left (*p* < 0.05).
Accidents	Kwon et al., 2021; Korea [59]	A survey was conducted on 441 students to explore the usage pattern in terms of frequency of use while walking, history of accidents, and apps used while walking.	Of 441 students, 95.9% used smartphones ‘sometimes’ or more often while walking. 91.8% and 54.6% of 423 used their phones while waiting for a signal and while crossing the crosswalk, respectively. 57.9% experienced accidents or near misses when using smartphones while walking. Text messaging (87.7%) was the most frequently used app while walking, followed by music and phone calls.
	Kim et al.,2017; Korea [15]	A total of 608 college students completed an online survey that included their experience of accidents (total number; traffic accidents; falls/slips; bumps/collisions; being trapped in the subway, impalement, cuts, and exit wounds; and burns or electric shocks), their use of a smartphone, the type of smartphone content they most frequently used, and other variables of interests. Smartphone addiction was estimated using Smartphone Addiction Proneness Scale, a standardized measure developed by the National Institution in Korea.	Compared with normal users, participants who were addicted to smartphones were more likely to have experienced any accidents (OR = 1.90, 95% CI: 1.26–2.86), falling from height/slipping (OR = 2.08, 95% CI: 1.10–3.91), and bumps/collisions (OR = 1.83, 95% CI: 1.16–2.87). The proportion of participants who used their smartphones mainly for entertainment was significantly high in both the accident (38.76%) and smartphone addiction (36.40%) groups.
3.3 Effects on academic performance	Han et al., 2019; Korea [60]	The authors investigated the effects of smartphone use by college students on their perceived academic performance.	The results showed a statistically significant relationship between smartphone addiction and academic performance (*p* < 0.05).
	Winskel et al., 2019; Korea [61].	This study examined the relationship between smartphone use during the study, problematic smartphone uses, and academic performance in Korean and Australian university students. 119 Korean and 270 Australian students aged between 18 and 26 years completed a survey comprised of a smartphone usage questionnaire, smartphone addiction scale, and self-report of their current Grade Point Average (GPA) score.	Average smartphone use and problematic smartphone use were found to be significantly higher for Korean compared to Australian students (*p* < 0.001). The more time a student spends using their smartphone, the more at risk they are for problematic smartphone use and possible academic performance costs.
	Lee and lee, 2020; Korea [62]	This study addresses the relationship between mobile device use and academic performance through three different models by controlling demographic data, technological infrastructure conditions, and daily total multi-tasking time.	The study found that an increase in the daily total mobile device use time negatively affected GPA (*p* < 0.05).
	Lee et al., 2015; Korea [2].	The authors carried out a cross-sectional study on 210 university students focusing on the level of university students’ addiction to their smartphones and to understand the difference between self-regulated learning, and learning flow, based on smartphone addiction level.	Results demonstrated that the higher the addiction level is, the lower level of self-regulated learning the students have (*p* < 0.05). Authors concluded that smartphone addict—learners were constantly interrupted by the other applications on their phones when they were studying and did not have enough control over their smartphone learning plan and process.
Kwon et al., 2018; Korea [63]		Between December 2015 and February 2016, the authors conducted face-to-face interviews with 12 university students with self-reported ADHD symptoms.	The results show that university students with ADHD symptoms had difficulties coping with repeated cycles of negative thoughts and worries, irregular lifestyles due to poor time management, dissatisfaction with academic performance and interpersonal relationships, self-dissatisfaction, and decreased self-esteem.
3.4 Procrastination and impulsivity	Jeong and Baek, 2015; Korea [64]	For this study, it conducted the questionnaire with 348 college students who showed higher use of smartphones and analyzed the data from the questionnaire.	It was found that smartphone addiction had significant influences on the adjustment to college life. As a result of examining the moderating effects of impulsivity in the relationship between smartphone addiction and adjustment to college life, there were moderating effects of impulsivity (*p* < 0.05).
3.5 Self-esteem, reduced social interaction, solitude and suicide	Kim et al., 2017; Korea [65].	Between 15 and 31 May 2016, 263 subjects completed a questionnaire consisting of questions on smartphone addiction, communication ability, loneliness, and the health of interpersonal relationships.	The smartphone addiction and human relationship health results were negatively correlated (*p* = 0.011). In contrast, communication ability and human relationship health results were positively correlated (*p* < 0.001). However, loneliness and human relationship health were not significantly correlated. The final multiple regression model explaining human relationship health included smartphone addiction (*p* = 0.006), communication ability (*p* < 0.001), and motivation for smartphone purchase (*p* = 0.035) as independent factors.
	Yoo et al., 2020; Korea [13].	The authors investigated the effects of empathy, self-control, and smartphone dependency on the interpersonal relationships of nursing students. The survey data was collected from 157 students through self-report questionnaires from 27 May 2019, to 31 May 2019.	Smartphone dependency (*p* < 0.001) showed significant differences according to smartphone use. Age was found to have a statistically positive correlation with self-control (*p* = 0.015) and it had a negative correlation with smartphone dependency (*p* = 0.005). Self-control was found to have a statistically positive correlation with empathy (*p* < 0.01), interpersonal relationships (*p* < 0.001), and a negative correlation with smartphone dependency (*p* < 0.001).
	Mira Son; 2018; Korea [66].	The authors, purposed to identify the levels of self-awareness, other-awareness, interpersonal relation competence, and smartphone and Internet addiction and identify the influence of self-awareness, other-awareness, and interpersonal relations competence on smartphone and Internet addiction in 479 nursing students who completed structured self-report questionnaires.	The participants’ smartphone addiction risk was shown to be 24.2%. Among the predictors, gender (*p* < 0.05), academic year (*p* < 0.01), self-awareness (*p* < 0.05) other awareness (*p* < 0.001), and interpersonal relation competence (*p* < 0.001) had significant influences on smartphone addiction.
	Jeong et al., 2020; Korea [67].	Secondary data from the 2017 Community Health Survey, a large-scale sample survey conducted yearly in South Korea, were analyzed for 190,066 adults over 19 years of age.	Approximately 21,345 (11.23%) of the 190,066 participants reported experiencing impairments in daily activities due to Internet, gaming, or smartphones (IGS) overuse at least once in the previous year and the impairments were more severe in males than females. Participants experiencing impairments in daily activities contacted their friends a significantly higher number of times (4 times or more per month) and engaged in leisure activities more frequently (more than once per month) than those without impairments. There was also a significant positive relationship between IGS overuse and stress, depression, suicidal ideation, and suicide attempts (95% CI = 1.549–2.571, *p* < 0.0001).
	Lee et al., 2018; Korea [68]	A cross-sectional study was used to perform a detailed analysis of smartphone addiction subscales and social support related to interpersonal competence of nursing students in 324 college students in Korea from February 2013 to March 2013.	Cyberspace-oriented relationship, which is a smartphone addiction subscale, and social support were positively correlated with the interpersonal competence of nursing students (1.360 (*p* = 0.004) and 0.555 (*p* < 0.001), respectively.)

Note: Study details include the study design and objective of the study.

## Data Availability

This is an evidence synthesis study all articles used in this systematic review are available online from primary studies.
